# Proteomics-based prognostic signature and nomogram construction of hypoxia microenvironment on deteriorating glioblastoma (GBM) pathogenesis

**DOI:** 10.1038/s41598-021-95980-x

**Published:** 2021-08-26

**Authors:** Ya-Dan Wen, Xiao-San Zhu, Dong-Jie Li, Qing Zhao, Quan Cheng, Yun Peng

**Affiliations:** 1 Department of Clinical Pharmacology, Xiangya Hospital, Central South University, 87 Xiangya Rd., Changsha, 410008 Hunan People’s Republic of China; 2 Institute of Clinical Pharmacology, Hunan Key Laboratory of Pharmacogenetics, Central South University, 110 Xiangya Rd., Changsha, 410008 Hunan Province People’s Republic of China; 3 Engineering Research Center of Applied Technology of Pharmacogenetics, Ministry of Education, 110 Xiangya Rd., Changsha, 410008 Hunan People’s Republic of China; 4 National Clinical Research Center for Geriatric Disorders, 87 Xiangya Rd., Changsha, 410008 Hunan People’s Republic of China; 5 Nanjing Yaning Biological Technology Co. Ltd, Najing, 211200 Jiangsu People’s Republic of China; 6 Shenzhen Runhua Zhilian Technology Co., LTD, Shenzhen, 518126 Guangdong People’s Republic of China; 7 Department of Gastroenterology, Chenggong Hospital, Xiamen University, 96 Wenyuan Road, Xiamen, 361003 Fujian People’s Republic of China; 8 Department of Geriatric Urology, Xiangya International Medical Center, Xiangya Hospital, Central South University, Changsha, 410008 Hunan People’s Republic of China; 9 Department of Geriatrics, Xiangya Hospital, Central South University, 87 Xiangya Rd., Changsha, 410008 Hunan People’s Republic of China; 10 Teaching and Research Section of Clinical Nursing, Xiangya Hospital of Central South University, Changsha, 410008 Hunan People’s Republic of China; 11 Department of Neurosurgery, Xiangya Hospital, Central South University, Changsha, 410008 Hunan People’s Republic of China

**Keywords:** Cancer, Computational biology and bioinformatics, Genetics, Neuroscience

## Abstract

The present study aimed to construct and evaluate a novel experiment-based hypoxia signature to help evaluations of GBM patient status. First, the 426 proteins, which were previously found to be differentially expressed between normal and hypoxia groups in glioblastoma cells with statistical significance, were converted into the corresponding genes, among which 212 genes were found annotated in TCGA. Second, after evaluated by single-variable Cox analysis, 19 different expressed genes (DEGs) with prognostic value were identified. Based on λ value by LASSO, a gene-based survival risk score model, named RiskScore, was built by 7 genes with LASSO coefficient, which were *FKBP2, GLO1, IGFBP5, NSUN5, RBMX, TAGLN2* and *UBE2V2*. Kaplan–Meier (K–M) survival curve analysis and the area under the curve (AUC) were plotted to further estimate the efficacy of this risk score model. Furthermore, the survival curve analysis was also plotted based on the subtypes of age, IDH, radiotherapy and chemotherapy. Meanwhile, immune infiltration, GSVA, GSEA and chemo drug sensitivity of this risk score model were evaluated. Third, the 7 genes expression were evaluated by AUC, overall survival (OS) and IDH subtype in datasets, importantly, also experimentally verified in GBM cell lines exposed to hypoxic or normal oxygen condition, which showed significant higher expression in hypoxia than in normal group. Last, combing the hypoxia RiskScore with clinical and molecular features, a prognostic composite nomogram was generated, showing the good sensitivity and specificity by AUC and OS. Meanwhile, univariate analysis and multivariate analysis were used for performed to identify variables in nomogram that were significant in independently predicting duration of survival. It is a first time that we successfully established and validated an independent prognostic risk model based on hypoxia microenvironment from glioblastoma cells and public database. The 7 key genes may provide potential directions for future biochemical and pharmaco-therapeutic research.

## Introduction

Tumor hypoxia, is characterized by insufficient oxygenation to support tumor growth and propagation, exacerbating malignancy of solid tumors^1^. Hypoxia tumor microenvironment leads to compensatory reactions by tumors, including chronic hypoxia response and acute hypoxia response^2^. Chronic hypoxia response, also called diffusion-limited hypoxia, generated to contributed to long-term tumor changes, like elevated tissue acidity, accumulation of DNA replication errors and high frequency of DNA breaks; acute hypoxia response, referred as perfusion-related hypoxia, contributed to high redox reaction and strong autophagy, leading to genomic instability from delayed DNA damage, spontaneous metastasis and aggressive radio-resistance^2^. The proteomic and genomic transformations of tumor adapted to oxygen starvation, gave rise to evolutionary selections of more malignant cancer phenotypes and was heavily resistant to curative treatments, no matter the therapy modality employed^3^.

Glioblastoma multiforme (GBM), among the primary brain tumor, was the most aggressive and malignant tumor with 15–17 months median survival rate^4^. GBM proliferated rapidly, leading to the inadequate vascularization and poor oxygen supply^5^. The variation of oxygen tension, as an important tumor microenvironment factor, was associated with heterogeneous regions presenting as different subpopulations of glioblastoma cells^6^. Hypoxia level in GBM was associated with the WHO grading of gliomas, i.e. grade 2 tumors being related with gentle hypoxia, while grade 4 tumors being correlated with severe cellular hypoxia^7^. Emerging evidences showed insufficient oxygen supply resulted in enhanced malignancy of GBM, presenting as instability of the genome, immunogenicity, chemo/radio therapy resistance, and high metastasis tendency^8^. Current studies regarding to the mechanisms of the hypoxia in GBM found increased expression of CD133 (a cancer stem cell marker), OCT4, STAT3 (an autophagy key protein) and VEGF (a migration factor), IL-1β (an apoptosis inducer) a^9, 10^. These studies did not help us to find any methods to predict patient survival and unveil the key genes which may be the potential direction of GBM pathological mechanisms. Therefore, it is an urgent need to develop a novel approach to further understand GBM, which will be beneficial for patient therapies.

To fill this gap, we successfully constructed an efficient, prognostically significant model composed of seven-mRNAs hypoxia signatures to assess the prognosis of GBM. These selected mRNAs were collected from previous proteomics data, which was identified 2,348 quantifiable proteins with 426 proteins having altered abundance (FDR < 0.05, fold change > 1.2 or < 0.83) and 62 proteins having significantly altered abundance (FDR < 0.05, fold change > 1.5 or < 0.67), including 28 up-regulated proteins and 34 down-regulated, in the normal and hypoxic glioblastoma LN18 cells. This is the first time to combine in vitro studies, proteomics analysis, bioinformatic analysis and clinical biological data to establish a hypoxia risk model, which has the potential value to assist researchers and clinicians to decide the personalizing treatments for glioblastoma patients.

## Materials and methods

### Analysis overview

**Figure 1:**
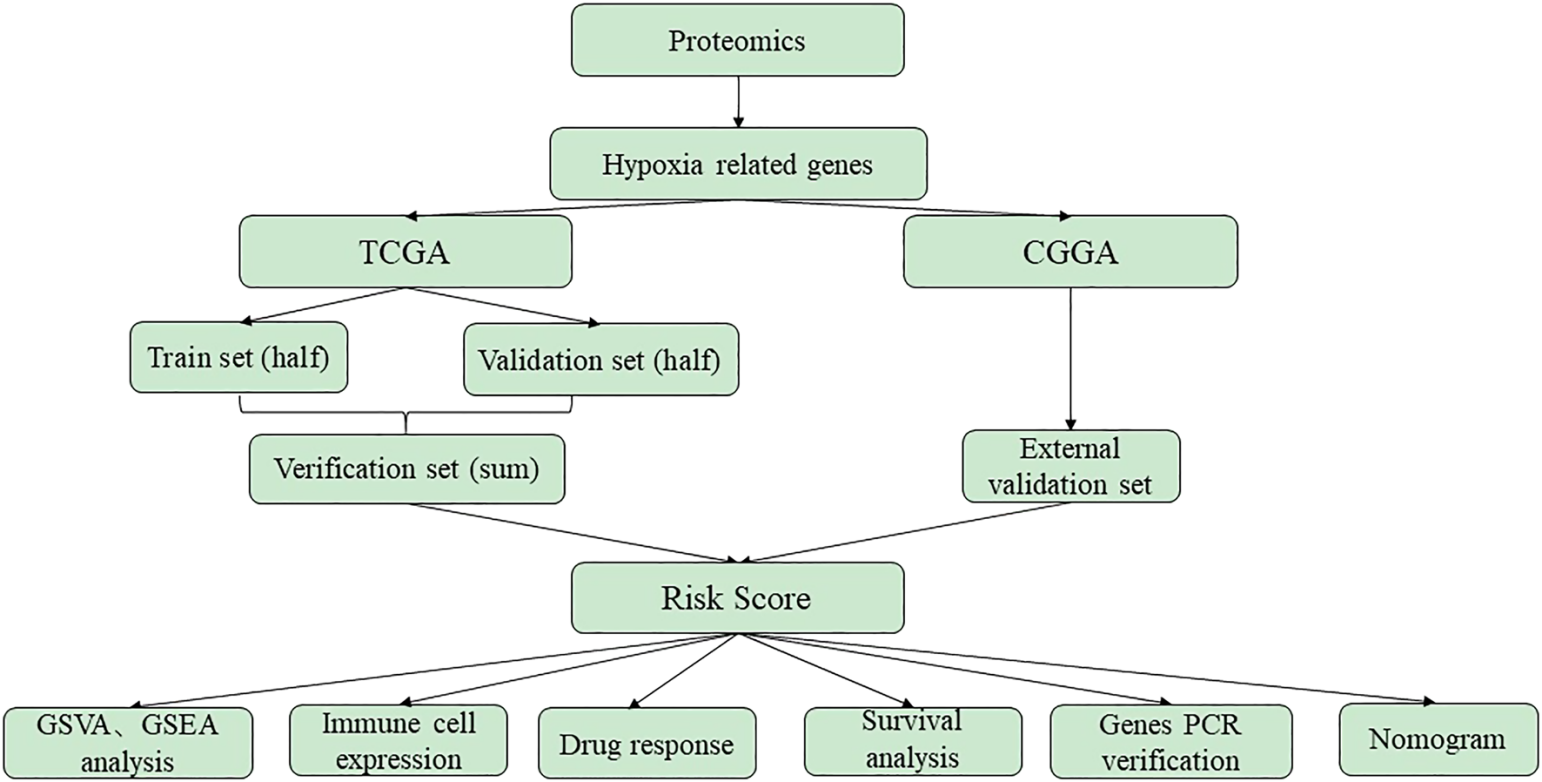
The workflow of generating proteomics-based hypoxia GBM risk score model.

In this study, the proteomics data was collected from glioblastoma LN18 cells under control and hypoxia treatments. The differentially expressed genes (DEGs) obtained from proteomics analysis were intersected with the genes in the “TCGA Glioblastoma (GBM)” of TCGA (https://xenabrowser.net/datapages/) and CGGA (http://www.cgga.org.cn). In the training set of TCGA, a univariate Cox scale risk regression analysis was performed using the survival package in R (version 3.6) to study the relationship between the overall survival (OS) of patients and the level of gene expression. The p value < 0.05 of a gene was considered having significant prognostic potential. Next, L1 punishment (Lasso) regression, a useful way to determine the predictable rules in high-dimensional data, was applied to further identify pseudogenes with independent prognosis value^10^. Based on the highest λ value selected through 1,000 cross-validations in the Lasso method, a set of prognosis genes and their LASSO coefficients were obtained^11^. LASSO coefficients were used to establish a gene-based survival risk assessment model. Patients were divided into low-risk and high-risk groups through the median risk score. Kaplan–Meier graph and Log-rank tests estimated and compared the OS of patients between the two risk groups. Time-dependent receiver operating characteristic (ROC) curves and AUC were used to evaluate the predictive accuracy of the risk model and the selected genes^12^. Gene Set Enrichment Analysis (GSVA), Gene Set Variation Analysis (GSEA) analysis, immune cell expressions, chemotherapy drug response and nomogram were evaluated. Finally, the individual hypoxic genes were assessed OS and expressions in datasets, and experimentally verified their expressions in hypoxic or normal GBM cell lines. The processing flow was shown in Fig. 1.

### TCGA and CGGA database preparation

Gene expression and appropriate clinical information were downloaded from the TCGA data source (TCGA GBM, AffyU133a microarray) and the CGGA data portal (CGGA RNAseq, batch 1). TCGA and CGGA data do not include samples that have survived for less than 30 days. The TCGA GBM (chip) group is randomly divided into two equal parts: the training set (train, set 1), and the validation set (test, set 2). The total TCGA GBM data is used as another validation set (sum, Group 3), while the CGGA GBM group is used as an external validation set (Group 4) in the following studies.

### Constitution and validation of the prognostic risk score model

Hypoxia genes found to be statistically significant in univariable Cox regression were then used lasso regression to achieve the coefficients; the risk-score formula was constructed as:

$$riskscore = \sum\limits_{{{\text{i}} = 1}}^{{\text{N}}} {\left( {{\text{Exp}}_{{\text{i}}} \times {\text{Coe}}_{{\text{i}}} } \right)}$$

where N = 7, Exp_i_ was the expression value of every seven hypoxia genes, and the Coe_i_ was the corresponding multivariable Cox regression coefficient^6, 13^.

The least absolute shrinkage and selection operator (L1-penalized, Lasso) Cox regression analysis is ideal to determine interpretable prediction rules in high-dimensional data^14^. LASSO Cox regression model were performed to determine the optimal coefficient and to calculate the deviance likelihood. The coefficients and deviance were calculated with the “glmnet” package in R. Based on the highest lambda value that was selected through a 1,000 cross-validations in Lasso method (lambda.min), a set of prognostic genes and their LASSO coefficients were obtained. According to coefficient, the datasets of TCGA and CGGA were divided into high- and low-risk subgroups based on the median risk scores. The prognostic model for OS was calculated by multiplying the expression level of each hypoxia-GBM genes and corresponding coefficient.

### Survival analysis

Kaplan–Meier (K–M) survival curve analysis was performed to further estimate the associations between two different groups and OS; *p* < 0.05 was set as the cutoff. The patient was divided into low-risk and high-risk groups using the median of RiskScore as a threshold^15–17^. OS was compared between the high and low hypoxia risk groups via K–M analysis using the survival and survminer packages in R. A ROC curve was generated to validate the accuracy of the risk model in predicting the patients’ OS via the survivalROC R package^12^.

### Construction and validation of the nomogram

All the independent prognostic risk factors were identified by univariate and multivariate Cox regression analysis, then construct the prognostic nomogram so that the OS probability was assessed at 1, 2, and 3 years for patients with GBM by the “rms” package in R (https://cran.r-project.org/web/packages/rms/). The calibration curves were drawn using the rms R package to compare the predicted and actual OS.

### Gene set enrichment analysis (GSEA) and gene set variation analysis (GSVA)

GSEA helps to determine whether distinct sets of genes have significant differences using computational methods. GSEA analysis were used the software Pi package of R language. Differences were considered statistically significant at |NES|> 1, nominal *p* value < 0.05, and adjp < 0.25. GSVA package was utilized to calculate the expressions of GO and KEGG terms in TCGA and CGGA datasets. Correlation analysis was performed by the expression values of risk score and GO or KEGG term. The items with *p* < 0.05 and high correlation coefficient were selected.

### Prediction of chemotherapeutic response

The chemotherapeutic response for each of the GBM patients was predicted according to the public pharmacogenomic database, Genomics of Drug Sensitivity in Cancer (GDSC, www.cancerrxgene.org). The prediction of drug sensitivity (IC50) values was conducted using the R package “pRRophetic”^14^.

### Reverse transcription-quantitative polymerase chain reaction (RT-qPCR)

Total RNA was isolated from LN18 cells using TRIzol reagent (Invitrogen, Carlsbad, CA, United States). The cDNA was synthesized using HiScript III RT SuperMix for qPCR Kit (Vazyme Biotech, Nanjing, China). The cDNA was subsequently analyzed using ChamQ Universal SYBR qPCR Master Mix (Vazyme Biotech, Nanjing, China) and the ABI7500 system (Applied Biosystems, Foster City, CA, United States). The amplification program was as follows: initial denaturation step at 95 °C for 30 s, followed by 40 cycles at 95 °C for 5 s, and 60 °C for 30 s. The expression of *FKBP2, GLO1, IGFBP5, NSUN5, RBMX, TAGLN2* and *UBE2V2* were calculated relative to the internal reference gene, GAPDH, using the 2^−△△Ct^ method^18^. Primer sequences were shown in Supplementary Table 1.

### Statistical analysis

Statistical analyses and graphics were undertaken using R version 3.6. Significant quantitative differences in statistics between and among groups were calculated by the methods of two‐tailed t test and one‐way ANOVA, respectively. The chi-square test was used to analyze the correlation of the classified data. Survival curves were compared with the log-rank test. The HR and 95% CI were estimated through a univariable Cox regression. *p* < 0.05 was considered statistically significant.

## Results

### Data preprocessing and DEGs screening

First, the proteomics analysis harvested 426 proteins having altered abundance in hypoxia and normal glioblastoma LN18 cells, which could be referred to our group’s article^19^. The survival and risk-score calculations of the proteomics data in TCGA and CGGA were attached in the supplemental files. Then, 426 proteins were converted to corresponding genes. Among them, 212 differential genes were found annotations in patients with GBM in the TCGA database. At the meantime, genetic data of GBM patients were collected from the TCGA database and CGGA database. Then, the genetic data of GBM patients in TCGA was randomly divided into two parts, one as data analysis (train set), another as data validation (test set). The final screening results were verified again in all GBM patient data (sum set). Finally, chi-square test was processed in the random grouping, finding that the three groups of the most clinical characteristics had no significant difference, seen in Table 1.

**Table 1:** Clinicopathological variables of GBM patients in TCGA dataset.

	Train	Test	All	χ2	P
**Age**				0.737	0.692
< 65y	69.2%	65.6%	67.4%		
≥ 65y	30.8%	34.4%	32.6%		
**Gender**				0.134	0.935
Female	38.4%	40.0%	39.2%		
Male	61.6%	60.0%	60.8%		
**Subtype**				8.535	0.201
Classical	22.0%	33.2%	27.6%		
Proneural	28.0%	24.8%	26.4%		
Mesenchymal	30.8%	28.0%	29.4%		
Neural	19.2%	14.0%	16.6%		
**OS**				0.361	0.835
Alive	15.6%	17.6%	16.6%		
Death	84.4%	82.4%	83.4%		
**Chemo**				0.189	0.996
No	14.0%	15.2%	14.6%		
Yes	72.8%	72.4%	72.6%		
**Radio**				2.329	0.675
No	9.2%	10.8%	10.0%		
Yes	86.0%	86.8%	86.4%		
**IDH**				13.332	0.010
Wt	64.4%	77.2%	70.8%		
Mutant	6.4%	7.2%	6.8%		

The 212 annotated genes were identified by univariate Cox analysis and found 19 hypoxia-related DEGs: *ARL1, BST2, CBR1, EIF3M, F3, FKBP2, GLO1, HEXB, HSPB1, IGF5, LDHA, NDRG1, NSUN5, P4HA2, RBMX, S100A4, SEPT9, TAGL2, UBE2V2*.

### Construction and evaluation of the prognostic risk score model

**Figure 2:**
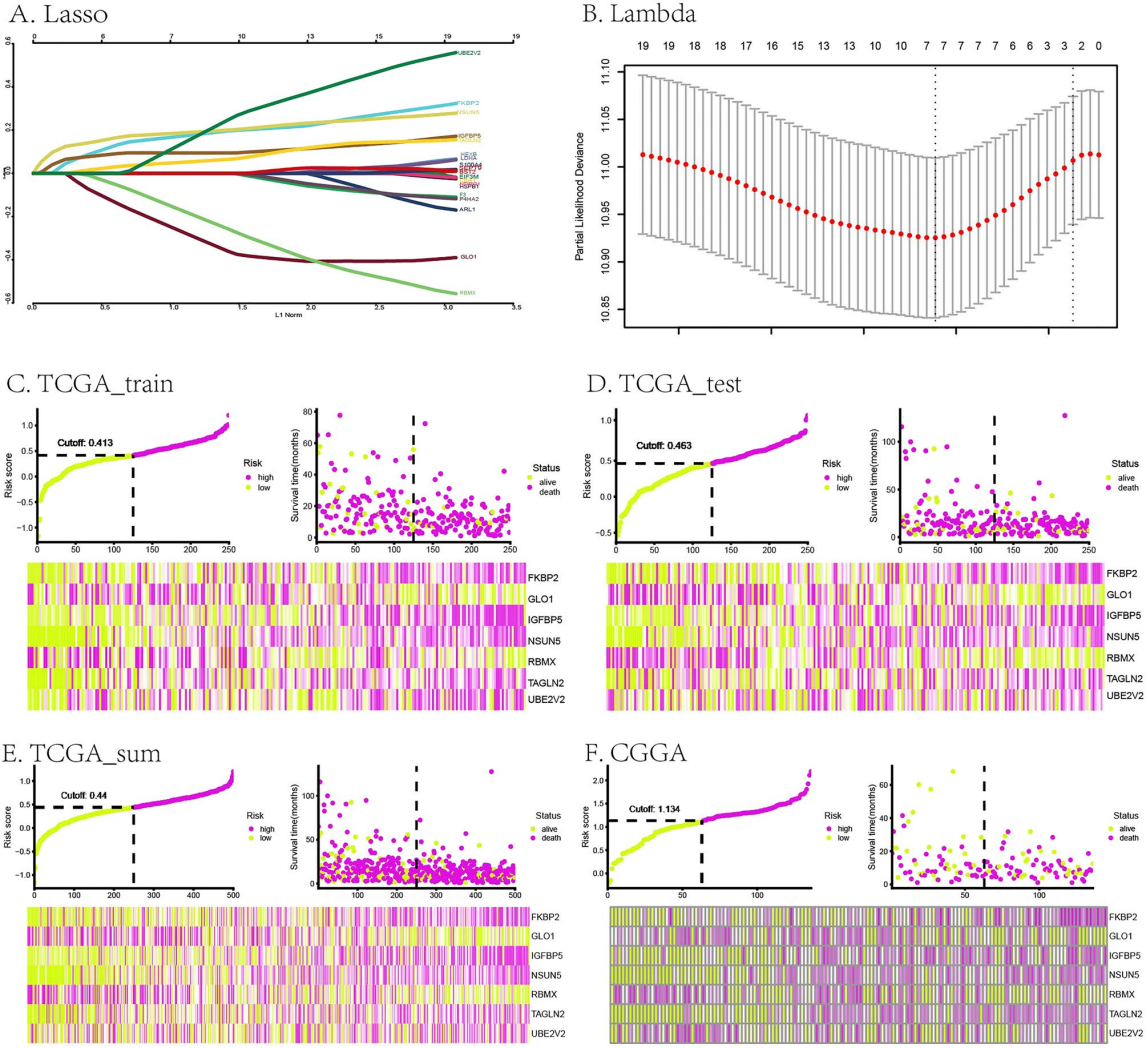
Construction of the prognostic risk score model based on a hypoxic 7-gene signature in GBM. (**A**,**B**) Lasso and Lambda of the risk score model. (**C**–**F**) risk score curves, dot diagrams and heat maps of the risk score model in TCGA (train set, test set, sum set) and CGGA.

The 19 hypoxia-related DEGs in GBM was further evaluated by Lasso regression analysis and 7 DEGs were left: *FKBP2, GLO1, IGFBP5, NSUN5, RBMX, TAGLN2, UBE2V2*. The prognostic risk model was established by RiskScore, which was the sum of multiplying the Lasso coefficients by the gene expression values of the seven genes: RiskScore = (0.18832677 * *FKBP2* expression level) + ((-0.3307785) * *GLO1* expression level) + (0.09428855 * *IGFBP5* expression level) + (0.19601453 * *NSUN5* expression levels) + ((-0.22900921) * *RBMX* expression level) + (0.06008254 * *TAGLN2* expression level) + (0.21064225 * *UBE2V2* expression level) . The Lasso and Lambda of this risk score model were shown in Fig. 2A,B. The green and purple curves in Fig. 2C–F represented the risk score curves. The green and purple dots diagrams in Fig. 2C–F indicated the distribution of patients’ survival status. The heat map in Fig. 2C–F showed the seven hypoxic signatures of every patient with GBM. The TCGA and CGGA riskscore and survival were seen in supplemental Table 2.

**Table 2:** Univariate and multivariate cox proportional hazards analysis of clinicopathological variables and hypoxia signature based on overall survival (OS) in the TCGA GBM cohort.

Characteristic	TCGA					
	*p*	HR	95%CI	*p*	HR	95%CI
	Univariate			Multivariate		
Riskscore	< 0.001	3.67	2.47–5.45	< 0.001	2.76	1.77–4.29
Age(≥ 65)	< 0.001	1.79	1.38–2.32	< 0.001	1.59	1.22–2.07
Gender (Male)	0.09	1.24	0.97–1.59	0.02	1.34	1.04–1.73
Subtype (Mesenchymal)	0.34	1.16	0.85–1.59			
Subtype (Neural)	0.65	1.08	0.76–1.54			
Subtype (ProneuRal)	0.09	0.75	0.54–1.05			
IDH(WT)	< 0.001	2.85	1.79–4.54	0.057	1.69	1.04–1.73
Chemotherapy (YES)	< 0.001	0.42	0.31–0.58	0.001	0.57	0.98–2.89
Radiotherapy (YES)	< 0.001	0.18	0.13–0.26	< 0.001	3.40	0.20–0.44

**Figure 3:**
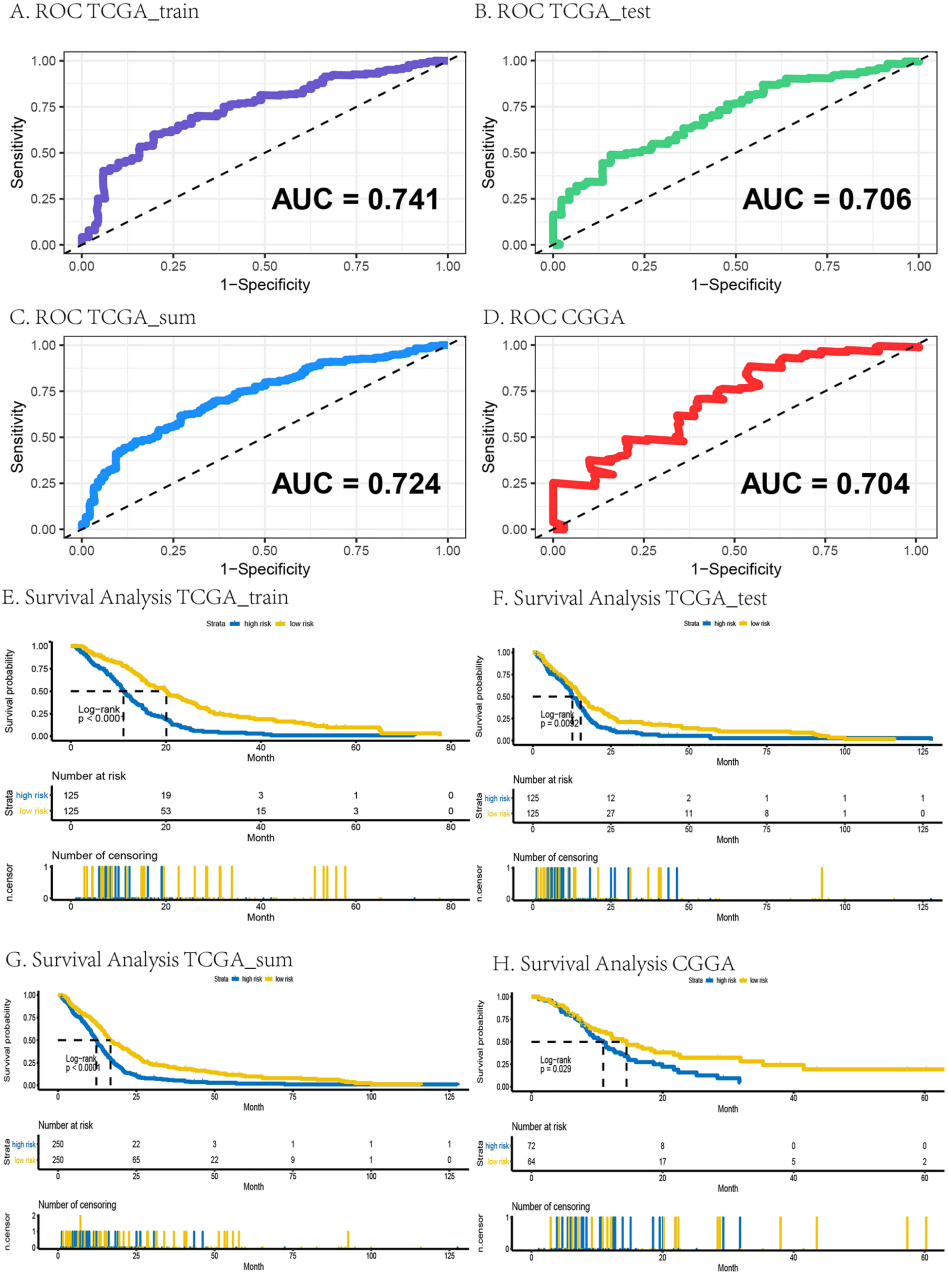
Verification of the prognostic risk score model based on a hypoxic 7-gene signature in GBM. (**A–D**) ROC curves in TCGA (train set, test set, sum set) and CGGA, respectively. (**E**–**H**) K–M survival curves in TCGA (train set, test set, sum set) and CGGA, respectively. The *p* values were shown in the plot respectively.

RiskScore was analyzed in the GBM data of train group, test group and sum group of TCGA database and CGGA database by ROC curve analysis. 4 generated AUCs of the RiskScore were higher than 0.7, indicating that the risk coefficient had high level of credibility, sensitivity, and specificity, shown in Fig. 3A–D.

According to the median RiskScore, the patients were divided into high and low risk subgroups, which were presented in composite graph of survival probability through K–M survival curve by GBM data of train group, test group and sum group of TCGA database and CGGA database. The significant separations of yellow and blue lines indicated that this prognostic risk model might distinguish between high and low risk patients, as shown in Fig. 3E–H (*p* < 0.05).

**Figure 4:**
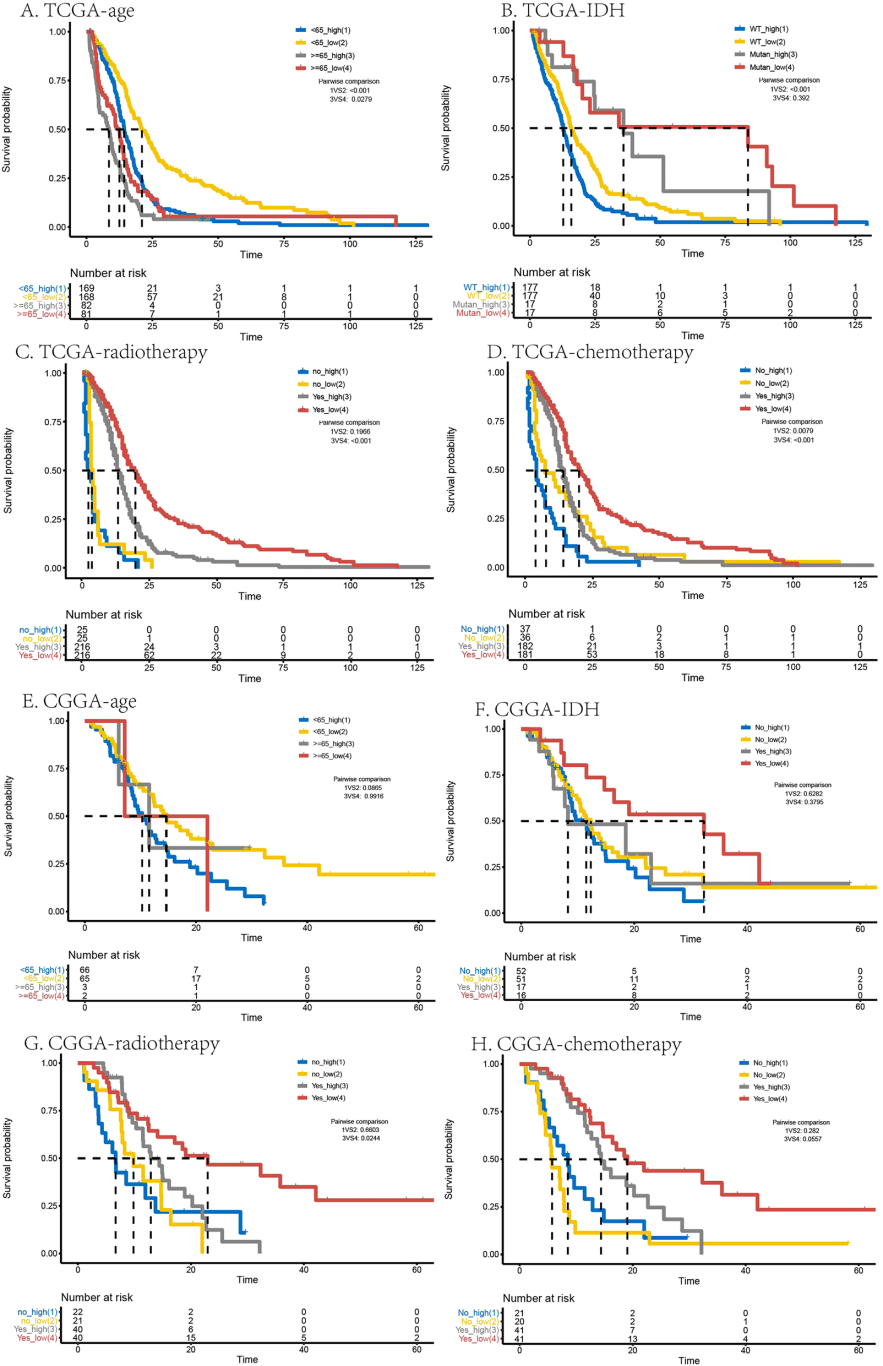
OS survival probability evaluated in GBM through age, IDH, radiotherapy, and chemotherapy in TCGA (**A**–**D**) and CGGA (**E–H**). The *p* values were shown in the plot respectively.

Additionally, the OS survival probability was also calculated by the K–M survival analysis in GBM by age (≥ 65 years or < 65 years) with high or low risk, IDH mutant or WT with high or low risk, radiotherapy (Yes or No) with high or low risk, and chemotherapy (Yes or No) with high or low risk in TCGA and CGGA sequence dataset, seen in Fig. 4A–H, respectively. Results of K–M survival analysis with above clinical features showed that low risk patients with less than 65 years old, IDH mutant, radiotherapy (Yes) and chemotherapy (Yes) had best survivals than other groups, indicating lower risk score, less than 65 years old, IDH mutant, radiotherapy (Yes) and chemotherapy(Yes) were the beneficial factors for survival.

### Key genes evaluated by survival curve and differential expressions

AUC of 7 genes selected from risk score model displayed good accuracy in TCGA datasets, especially NSUN5 and TAGLN2, as shown in Supplemental Fig. 1.

The median value of gene expression level was used for grouping, i.e., the expressions of higher than the median value were set as the high expression group, and the expressions of lower than the median value were the low expression group. The* p* values of survival probability curve between the high-expression and low-expression groups in *FKBP2, GLO1, IGFBP5, NSUN5, RBMX* and *TAGLN2*, were lower than 0.05, meaning that these genes may distinguish survival probability between the high and low expression groups, as shown in Supplemental Fig. 2.

**Figure 5:**
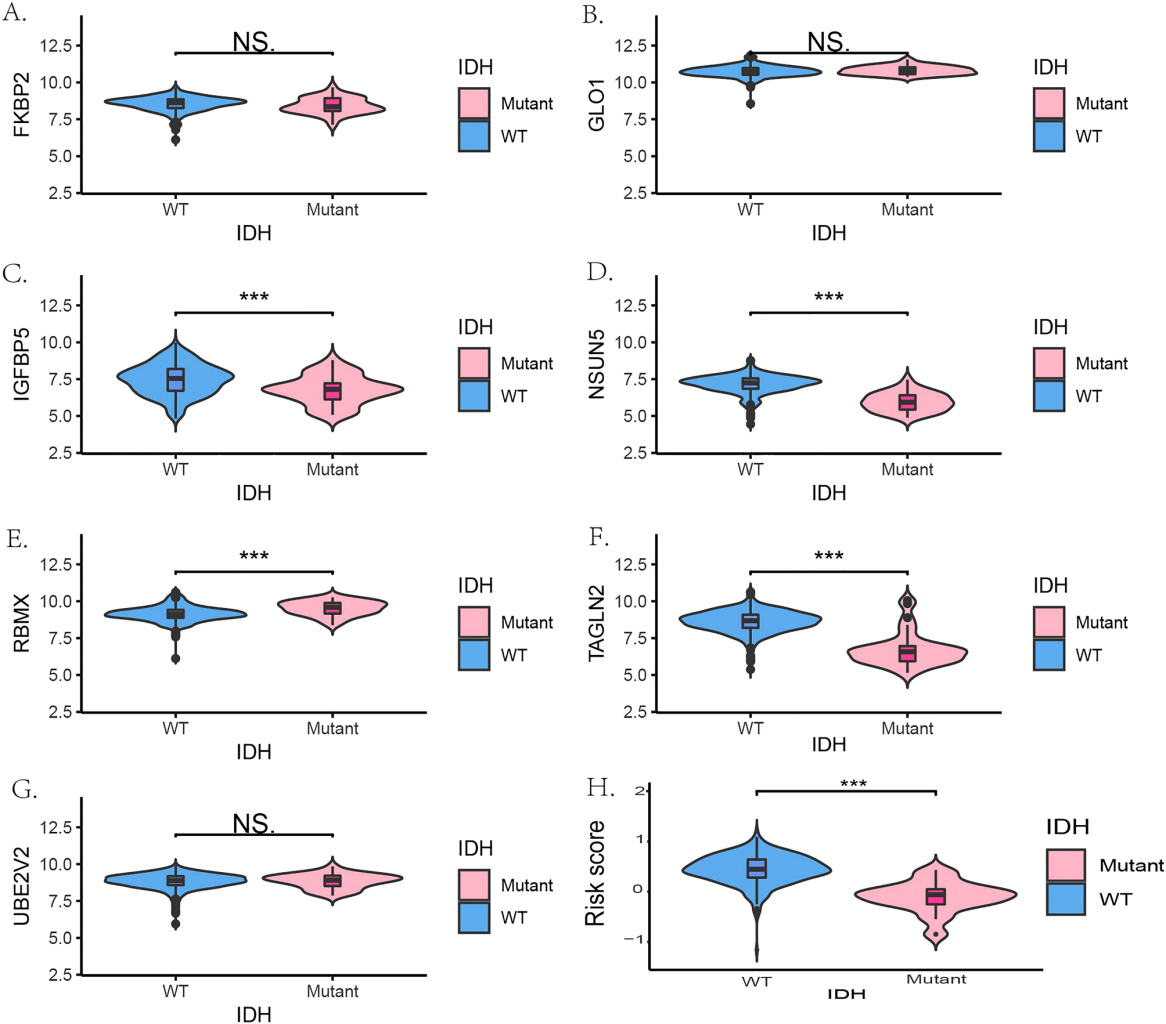
The gene expressions of seven genes between IDH WT and mutant patients in TCGA. (**A**–**G**) FKBP2, *GLO1, IGFBP5, NSUN5, RBMX, TAGLN2* and *UBE2V2*, respectively. ***p < 0.001. *WT* wild type.

As isocitrate dehydrogenase (IDH) mutant underwent heterozygous mutations in > 70% glioma patients, the relationships between gene expressions of these 7 hypoxia-related key genes and IDH mutant were investigated in TCGA and CGGA database. *IGFBP5, NSUN5, TAGLN2* and general risk score were dramatically expressed lower in IDH mutant patients than patients with IDH wild type (WT) in TCGA, and *FKBP2, IGFBP5, NSUN5, TAGLN2* and general risk score were dramatically expressed lower in IDH mutant patients in CGGA database, as shown in Fig. 5 and Supplemental Fig. 3, respectively.

To verify the gene expressions of 7 hypoxia-related DEGs screened from proteomics analysis in vitro and bioinformatics from TCGA and CGGA, GBM cell lines, LN18 cells, were suffered hypoxia and normal condition and analyzed relevant mRNA expression levels through RT-PCR. In LN18 cells, the gene expressions of *IGFBP5, RBMX, TAGLN2* and *GOL1* were changed dramatically in hypoxia compared to normal group (*p* < 0.05, Supplemental Fig. 4).

### Immune infiltration, GSVA, GSEA analyses and drug sensibility of risk score model

**Figure 6:**
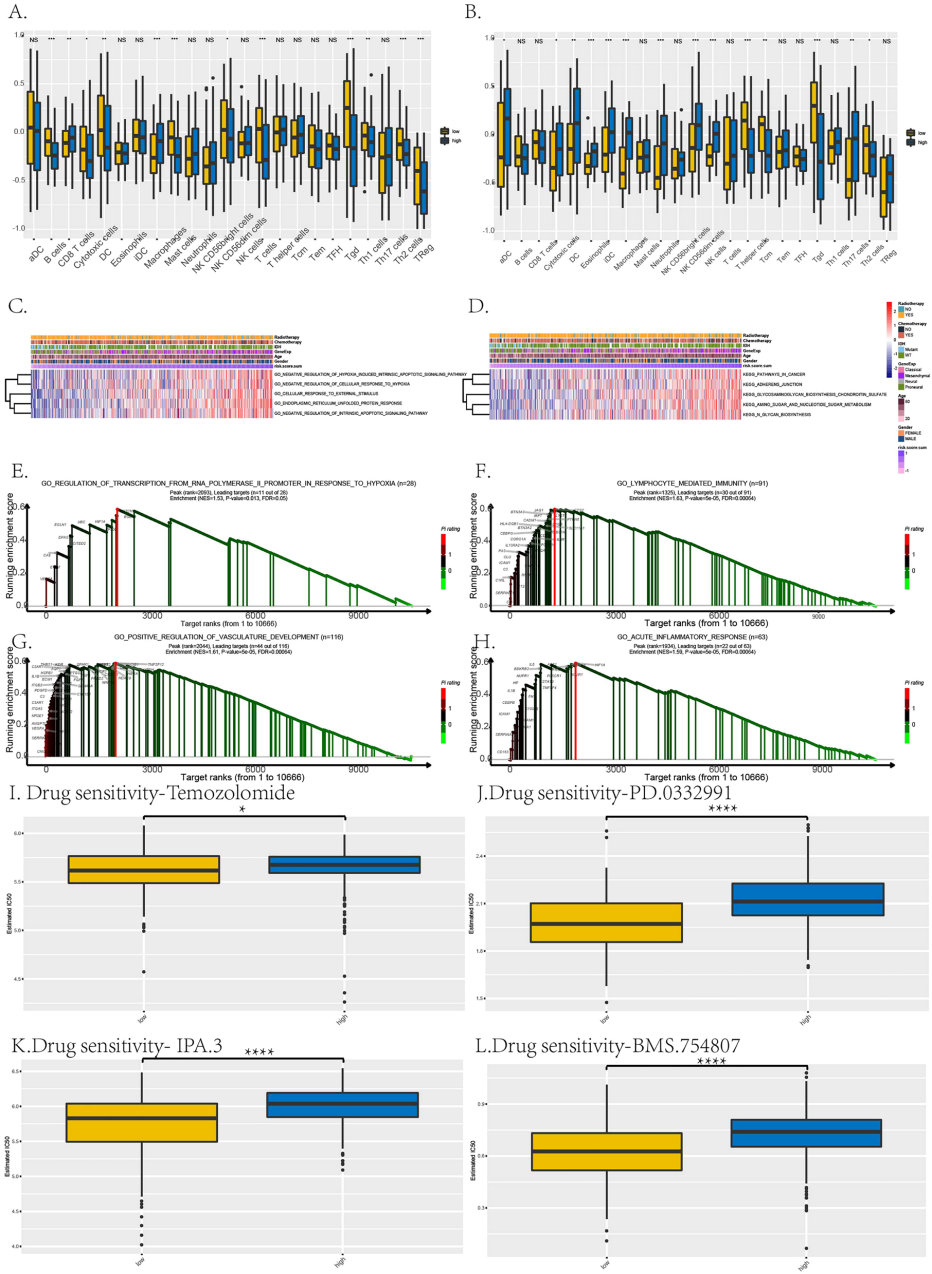
Immune infiltration, GSVA, GSEA analyses and prediction of chemotherapeutic response of the prognostic risk score model. (**A**,**B**) Immune cells expressed levels between high- and low-risk groups in TCGA (**A**) and CGGA (**B**). (**C**,**D**) The GSVA analysis in TCGA (**C**) and CGGA (**D**). (**E**,**H**) The GSEA analysis in TCGA. (**I**–**L)** Drug sensitivity of this risk score model, temozolomide, PD.0332991, IPA.3, and BMS.754807 as representatives. *p < 0.05; ****p < 0.0001.

Analyzing this RiskScore through immune infiltration, the immune cells, especially macrophages, were significantly expressed differently in high-risk group compared to low-risk group in TCGA and CGGA datasets, presenting the immunosuppression and malignancy in high-risk group of GBM (Fig. 6A,B).

GSVA revealed our 7-gene RiskScore was related to the GO pathways of hypoxia, endoplasmic reticulum unfolded protein response and apoptosis, and KEGG pathways of cancer, adherence junction and tumor metabolisms, shown in Fig. 6C,D. GSEA discovered that our hypoxia-based RiskScore could enriched in the GO pathways related to hypoxia, immunity, development of tumors vasculature and inflammatory response, shown in Fig. 6E–H.

As chemotherapy was the common treatment for GBM patients, the chemo drugs were analyzed the sensibility by estimated IC50 between high and low risk group, shown in Fig. 6I–L. Temozolomide, PD.0332991, BMS.754807 and IPA.3, as the representatives, showed that the drugs with significantly higher IC50 in high-risk group indicated worse drug sensitivities of these 4 drugs in high-risk group than low-risk group, based on our hypoxia-based RiskScore (*, *p* < 0.05; ****, *p* < 0.0001).

### Construction and evaluation of the clinical-featured risk model to the prognosis of GBM

To determine whether the prognostic significance of the hypoxic risk model was independent of other clinicopathological parameters in predicting the OS of GBM patients, univariate and multivariate Cox regression analyses were performed. Through HR and *p* value of risk score, age, gender, IDH, chemotherapy and radiotherapy in TCGA and CGGA indicated that the hypoxic risk model was significantly associated with OS, showing as the independent prognostic predictor, seen in Table 2 (TCGA) and Table 3 (CGGA).

**Table 3:** Univariate and multivariate cox proportional hazards analysis of clinicopathological variables and hypoxia signature based on OS in the CGGA GBM validation cohort.

Characteristic	CGGA					
	*p*	HR	95%CI	*p*	HR	95%CI
	Univariate			Multivariate		
Riskscore	0.01	1.96	1.16–3.32	0.075	1.69	0.95–3.02
Age(≥ 65)	0.92	1.06	0.39–2.90			
Gender (Male)	0.10	1.51	0.92–2.48	0.14	1.46	0.89–2.4
Subtype (Mesenchymal)	0.04	1.74	1.02–2.99			
Subtype (Neural)	0.29	0.59	0.22–1.57			
Subtype (ProneuRal)	0.93	1.03	0.54–1.95			
IDH (WT)	0.24	1.37	0.81–2.34			
Chemotherapy (YES)	< 0.001	0.32	0.21–0.51	< 0.001	0.40	0.25–0.64
Radiotherapy (YES)	< 0.001	0.40	0.25–0.64	0.002	0.48	0.30–0.77

**Figure 7:**
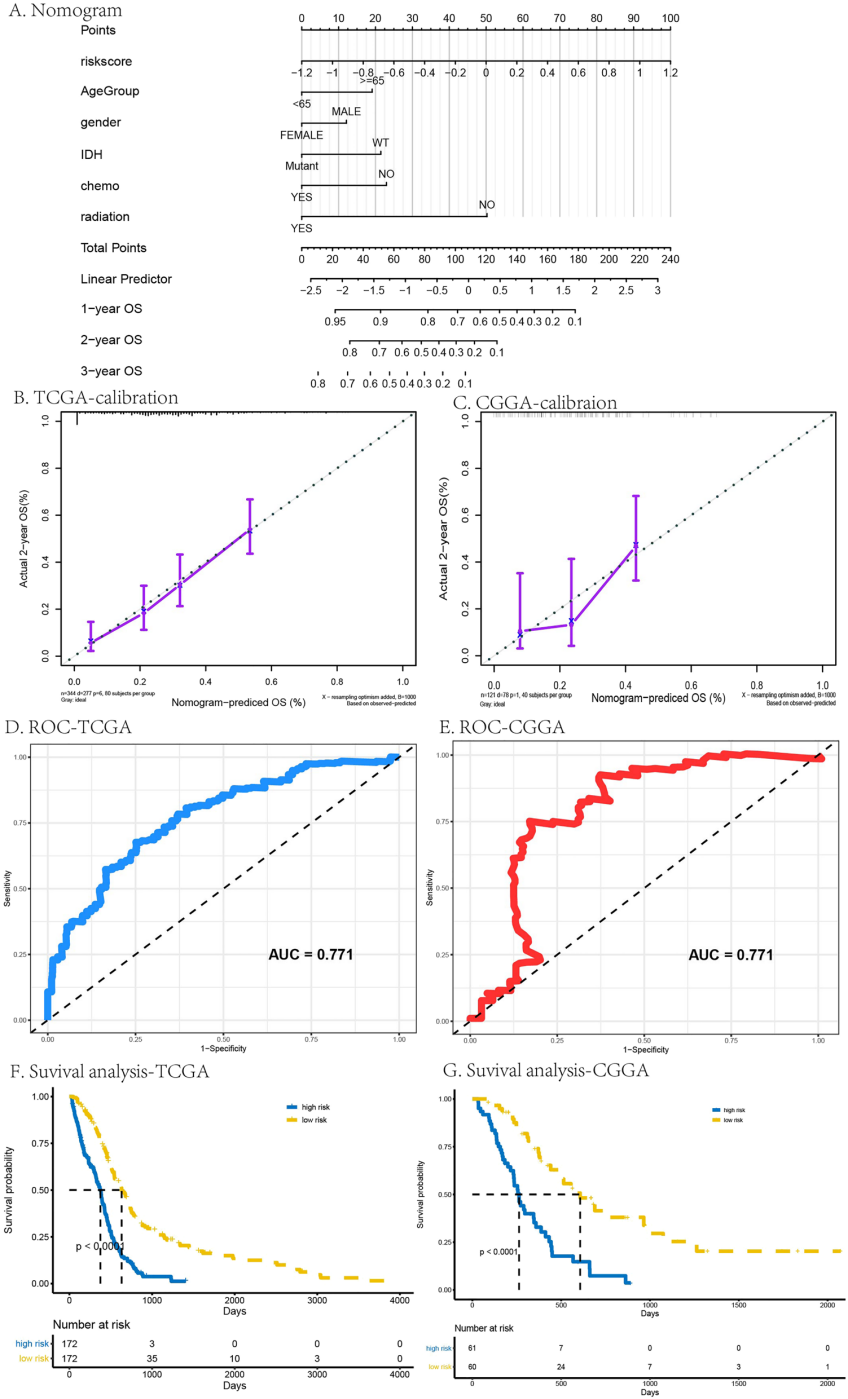
Construction and evaluation of the prognostic composite nomogram. (**A**) the prognostic nomogram to predict the OS probability at 1, 2, and 3 years based on the GBM patients in TCGA and CGGA. (**B**,**C**) The calibration plots in TCGA (**B**) and CGGA (**C**). (**D**,**E**) ROC curve analysis of the prognostic nomogram in TCGA (**D**) and CGGA (**E**). (**F**,**G**) K–M survival curve of the prognostic nomogram in TCGA (**F**) and CGGA (**G**). The *p* values were shown < 0.0001.

The prognostic composite nomogram was constructed to predict the OS probability at 1, 2, and 3 years based on the train group of patients with GBM, seen in Fig. 7A. The 5 significantly independent parameters, including age, gender, IDH, chemotherapy and radiotherapy, were recruited in the clinical-featured risk model. The calibration plots displayed excellent predict efficiency between probability and actual OS in train group of TCGA and CGGA database, seen in Fig. 7B,C. ROC curve analysis of this clinical-featured risk model showed high AUC, which was 0.771 both in TCGA and CGGA database, seen in Fig. 7D,E. K–M survival curve also validated the good performance of the clinical-featured risk model that differentiated the survival probability between high- and low-risk group, showing the good prognosis prediction (*p* < 0.05, Fig. 7F,G ).

### Construction and evaluation of the prognostic risk model based on validated mRNAs

According to PCR results, there were 3 genes in RiskScore consistently and individually showed significantly different expressions in normal hypoxia LN18 cells. Therefore, an attempt was made to generate another RiskScore to prognose GBM patients. RiskScore = ((− 0.4515390) * *GLO1* expression level) + (0.1738497 * *IGFBP5* expression level) + (0.1955157 * *TAGLN2* expression level).

RiskScore was analyzed in the GBM data of train group, test group and sum group of TCGA database and CGGA database by ROC curve analysis. 4 generated AUCs of the RiskScore were around 0.6, indicating that the risk coefficient had relative high level of credibility, sensitivity, and specificity, shown in Supple. Figure 5E-H.

According to the median RiskScore, the patients were divided into high and low risk subgroups, which were presented in composite graph of survival probability through K–M survival curve by GBM data of train group, test group and sum group of TCGA database and CGGA database. The significant separations of yellow and blue lines indicated that this prognostic risk model might distinguish between high and low risk patients, as shown in Supple. Figure 5A-D (*p* < 0.05).

## Discussion

Low tumor oxygenation, referred as hypoxia, was the major concern on the pathology of tumors, including GBM, a grade IV astrocytoma with great lethal and aggressive characteristics^4^. Current treatments of GBM, which were tumor resection surgery followed by chemo/radiotherapy, were dampened by gradual development of therapy resistance and tumor regrowth/relapse^4, 5, 20^. Therefore, there is a need to prognose the patients risk grade and survival to assist clinical treatments, based on the concerns that hypoxia was the deteriorated factor of heterogeneous tumor environment to promote the malignancy and migration. It could be an effective approach to construct a novel and prognostic model according to hypoxia risk genes through genetic profile analysis.

Recent developments of bioinformatics concepts and technologies have promoted the identification of genes in specific pathological mechanisms that permit researchers to target and further explore the potential molecular risk factors and predict GBM patients’ survival. It could be found 2 studies established the risk models from datasets for glioma. One study extracted hypoxia-related gene from GSEA constructed 5-gene prognostic signature by integrated data of lower-grade glioma (LGG) and GBM^6^. However, GBM, the grade IV astrocytoma, suffered different hypoxia, gene profiles and pathogenesis with LGG, the grade II and III astrocytoma, so that there is a necessary to separate the two diseases and build the prognosis model differently. Another study reported a 5-gene hypoxic signature of GBM from dataset without any experimental verification^21^. In our study, we firstly using proteomics from real hypoxic GBM samples not by identification of hypoxic genes in public datasets, constructed a 7-gene prognostic signature specific for GBM with good sensitivities. Additionally, our prognostic risk model possessed a stable predictive efficacy in 2 databases and potential risk genes were validated in GBM cell line. To better help clinicians to decide the treatments, chemo drugs sensitivity and immunosuppression were assessed. Moreover, patients were divided into the subtypes, including age, IDH, radiotherapy, chemotherapy to prognose their OS, so that clinicians could design the precision treatment for their patients based on the individual clinical features.

Our risk model was built on 7 hypoxia-related genes, of which 4 genes were validated the significantly different expressions in GBM cell lines under hypoxia stimulation compared to the normal treatment. Therefore, a RiskScore was built through these validated genes by PCR, which AUCs were around 0.6, lower than the RiskScore by 7 hypoxia-related genes. This indicated that the RiskScore by 7 hypoxia-related genes presented better prognosis of GBM.

Lastly, some genes such as FKBP2 and NSUN5 have good AUC and K–M survival curves but are not significantly changed as validated by RT-PCR. Because AUC and K–M survival curves had not direct correlation with gene expression difference. Additionally, *IGFBP5, RBMX* and *TAGLN2*, had consistently investigations in peers’ mechanical studies that the three genes were upregulated and promoted tumor metastasis. Due to the limited studies, *IGFBP5, RBMX* and *TAGLN2* were discussed scientific findings in relevant references and their different roles in hypoxic GBM, seen below.

*IGFBP5,* referred as insulin-like growth factor binding protein 5, is one of IGF binding proteins family compromised by six proteins that function as critical regulators of Insulin-like growth factor 1 (IGF-1) bioavailability^22–24^. In studies, IGFBP5 showed different regulated effects in cancers and metastatic tissues^22, 24^. Several studies found the dysregulation of tumor proliferation and metastasis, may due to a direct transcriptional target of PI3K-Akt-mTORC1 pathway^25^. However, Dong et. al reported in high grade glioma IGFBP5 up-expressed, and the knockdown IGFBP5 suppressed cell invasion in GBM cell lines U251 and one human GBM primary cell line^23^. Most recently, Rodvold et. al. investigated that IGFBP5 was overexpressed non-responders of chemotherapy; meanwhile, CRISPR-mediated deletion of IGFBP5 signature genes in the U251 human GBM cell line could elevate the response to chemotherapy^26^. These studies focused on GBM, the most malignant glioma, presented IGFBP5 was an oncogene, which was in line with our results that IGFBP5 was significantly higher in GBM patients and hypoxia GBM cell lines than control, showing sensitivity in prediction of GBM prognosis. Therefore, we have the reasons to advise *IGFBP5* may better stratify GBM patients and was the potent targets of mechanism study of GBM.

*RBMX*, recognized as an RNA binding protein that contributes to DNA double strand breaks^27^. This finding was identified by a genome-wide siRNA-based screen that RBMX was one of three candidate protein localized to the DNA damage^27^. It was also showed that *RBMX* was a heterogeneous nuclear ribonucleoprotein playing a role in alternative splicing, axial muscle segmentation and neural plate mispatterning^27, 28^. This pathological mechanism may be involved in multiple domains of a poly (ADP-ribose) polymerase 1-dependent manner so that RBMX could accumulate DNA damages, which may be through *BRCA2* expression^27, 28^. In our study, *RBMX* was significant expressed higher in hypoxic GBM samples and IDH mutant GBM patients in databases. Considering peers’ mechanical studies, our finding of higher expression of *RBMX* may be led by severe DNA damages under hypoxia, which need to be verified by future investigations.

*TAGLN2* (*SM22β*), the gene of transgelin-2, was translated to one of actin-bundling protein family with three isoforms that contained a calponin homolog (CH) domain^29^. Due to the actin-binding loop and calponin homolog domain, transgelin-2 could mechanically connect two adjacent actins, so that mediated multimeric interactions^29^. It was reported that transgelin-2 was the only transgelin family member expressed in immune cells, involving in the regulation of smooth muscle differentiation, lymphocyte activation, phagocytosis, tumor progression or metastasis, and mature synapse formation^30, 31^. Additionally, there was a report that *TAGLN2* was expressed higher in increasing tumor grade of glioma, worse OS; silenced TAGLN2 induced decreased invasion, cell arrest and apoptosis in vitro and inhibited tumorigenesis in vivo^32^. These investigations were in accordance with our results that *TAGLN2* has increasing expression in severe glioma (GBM) patients and worse tumor-hypoxic condition. Based on these findings, *TAGLN2* may be the potentially therapeutic target of GBM, waiting for relevant research to deeply explore.

Limitations inevitably impact on our study. First, although our genetic data was generated from GBM cells not from database, it would be more interesting if analyzed from human GBM patients. Second, there were 4 genes validated by in vitro hypoxia studies. It may be due to the variation of in vitro study and the difference between PCR and proteomics analysis. Additionally, it has the necessity that mechanisms underlying hypoxia-related genes involved in the pathogenesis of GBM should be further validated.

## Conclusion

It is the first time to develop and validate an experiment-based hypoxic prediction model for GBM. This hypoxia risk model was established on 7 genes reflecting immunity intensity of GBM microenvironment and serving as an independent prognostic factor of GBM patients. Our findings on hypoxia-based RiskScore of GBM could promote the precision therapy based on patients’ clinical features and the sensitivity of chemotherapy and radiotherapy and arouse more interests of researchers to target these genes and investigate novel mechanical pathways, so that potential pharmaco-therapies may be developed based on the gene profiling.

## Supplementary Information

Supplementary Information.

Supplementary Figure 1.

Supplementary Figure 2.

Supplementary Figure 3.

Supplementary Figure 4.

Supplementary Figure 5.

Supplementary Table 1.

Supplementary Table 2.

Supplementary Table 3.
