# Supplementary material for: Proteomics-based prognostic signature and nomogram construction of hypoxia microenvironment on deteriorating glioblastoma (GBM) pathogenesis
Source: Sci Rep. 2021 Aug 26;11:17170. doi: 10.1038/s41598-021-95980-x (PMC8390460; doi:10.1038/s41598-021-95980-x)
Supplement: Supplementary file 1 — Supplementary Information. [file 41598_2021_95980_MOESM1_ESM.docx]

Supple. Fig. 1. ROC of seven genes in TCGA dataset. A-G. FKBP2, GL01, IGFBP5, NSUN5, RBMX, TAGLN2 and UBE2V2, respectively.

Supple. Fig. 2. K-M survival curves of seven genes in TCGA dataset. A-G. FKBP2, GL01, IGFBP5, NSUN5, RBMX, TAGLN2 and UBE2V2, respectively. The *p* values of seven genes were shown the accurate number.

Supple. Fig. 3. The gene expressions of seven genes between IDH WT and mutant patients in CGGA dataset. A-G. FKBP2, GL01, IGFBP5, NSUN5, RBMX, TAGLN2 and UBE2V2, respectively. **, *p* < 0.01; ***, *p* < 0.001. WT, wild type.

Supple. Fig. 4. The gene expressions of seven genes by RT-PCR in LN18 cells under normal or hypoxia condition. A-G. FKBP2, GL01, IGFBP5, NSUN5, RBMX, TAGLN2 and UBE2V2, respectively. *, *p* < 0.05; ***, *p* < 0.001.

Supple. Fig. 5. Verification of the prognostic risk score model based on a hypoxic 3-gene signature in GBM. A-D. K-M survival curves in TCGA (train set, test set, sum set) and CGGA, respectively. E-H. ROC curves in TCGA (train set, test set, sum set) and CGGA, respectively. The *p* values were shown in the plot, respectively.

Supplementary Table 1. Primer sequences list used in RT-PCR

Supplementary Table 2. The TCGA and CGGA riskscore and survival.
