# Supplementary figures and images for: Proteomics-based prognostic signature and nomogram construction of hypoxia microenvironment on deteriorating glioblastoma (GBM) pathogenesis
Source: Sci Rep. 2021 Aug 26;11:17170. doi: 10.1038/s41598-021-95980-x (PMC8390460; doi:10.1038/s41598-021-95980-x)

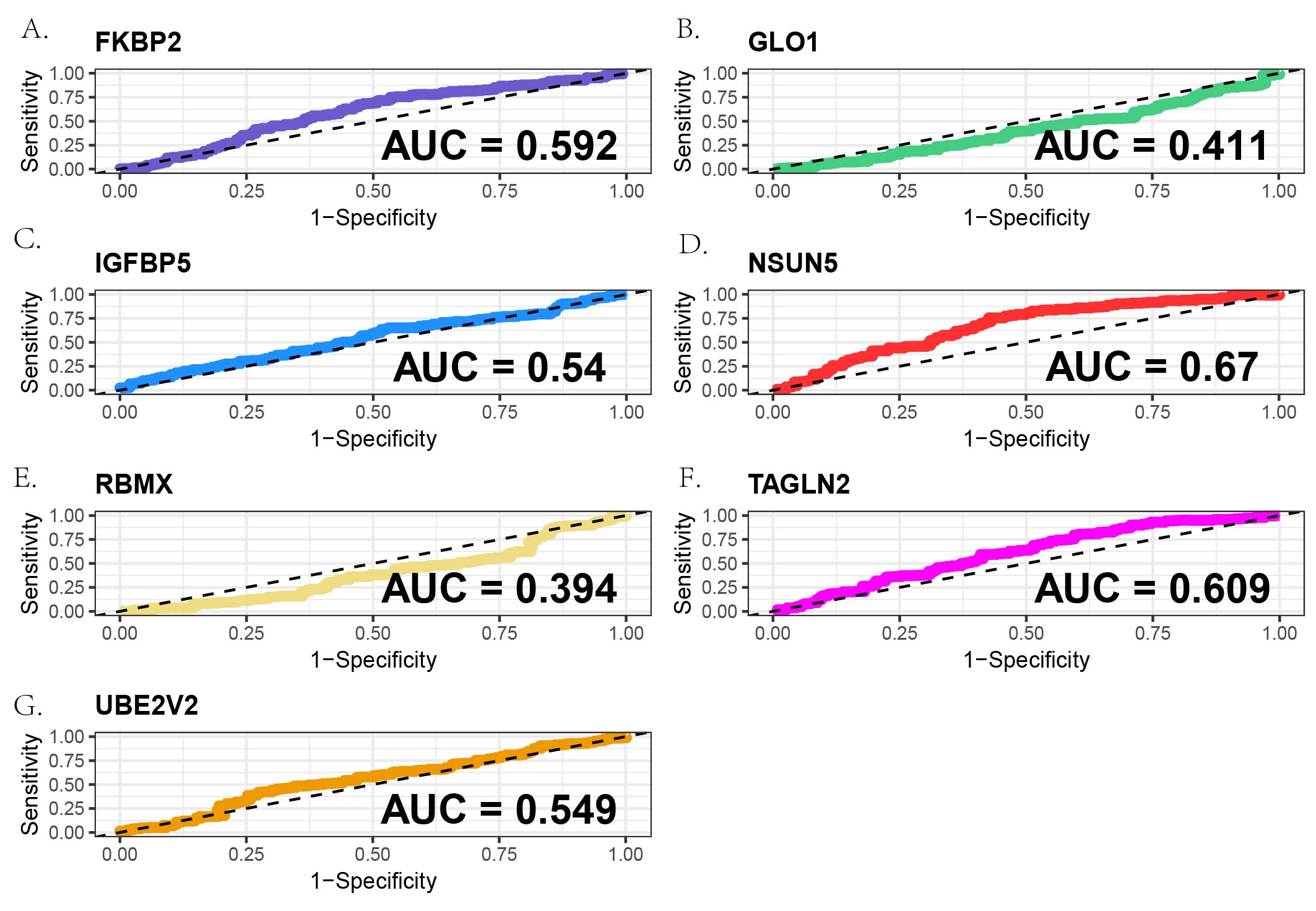

Supplement: Supplementary file 2 — Supplementary Figure 1. [file 41598_2021_95980_MOESM2_ESM.jpg]

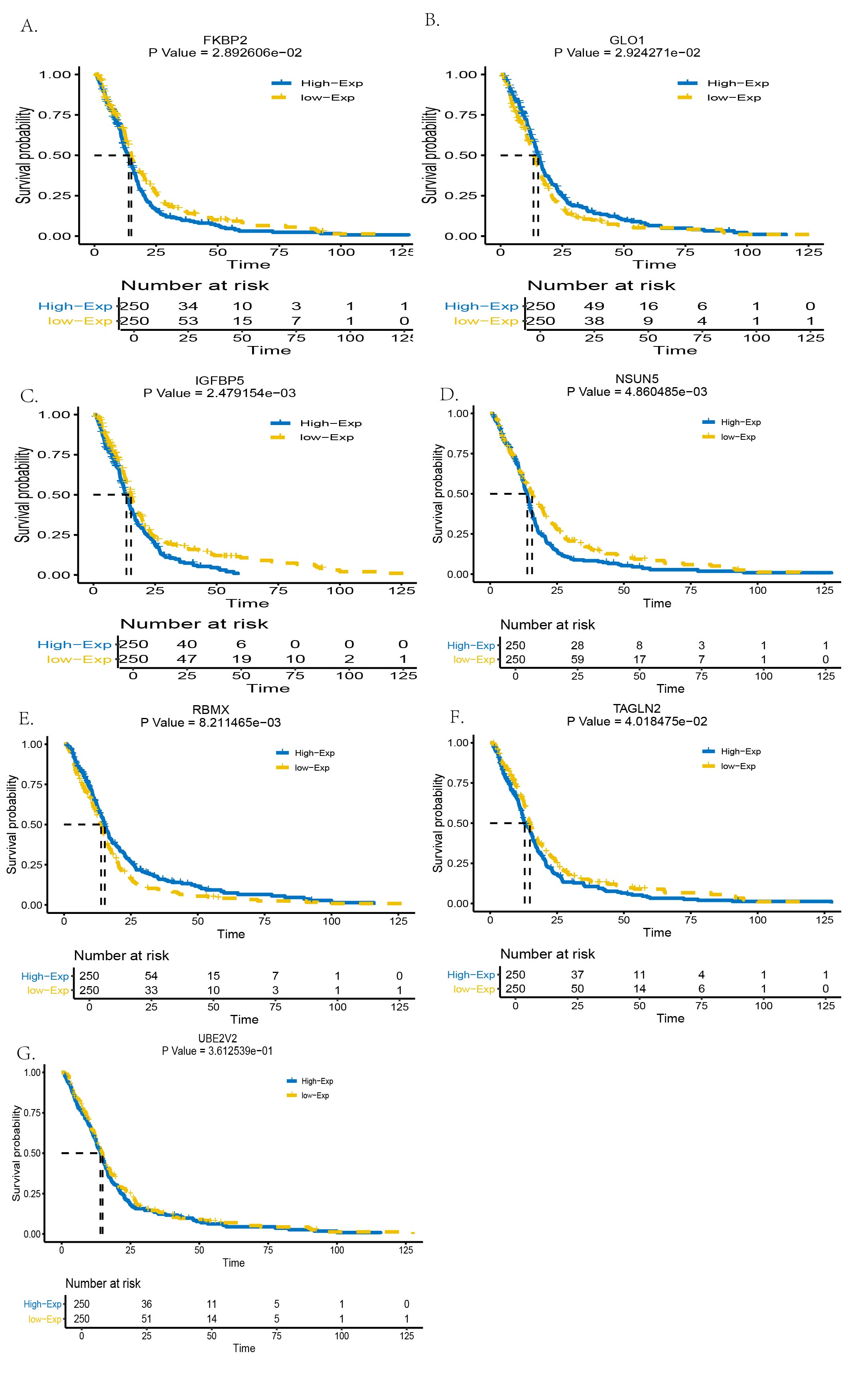

Supplement: Supplementary file 3 — Supplementary Figure 2. [file 41598_2021_95980_MOESM3_ESM.jpg]

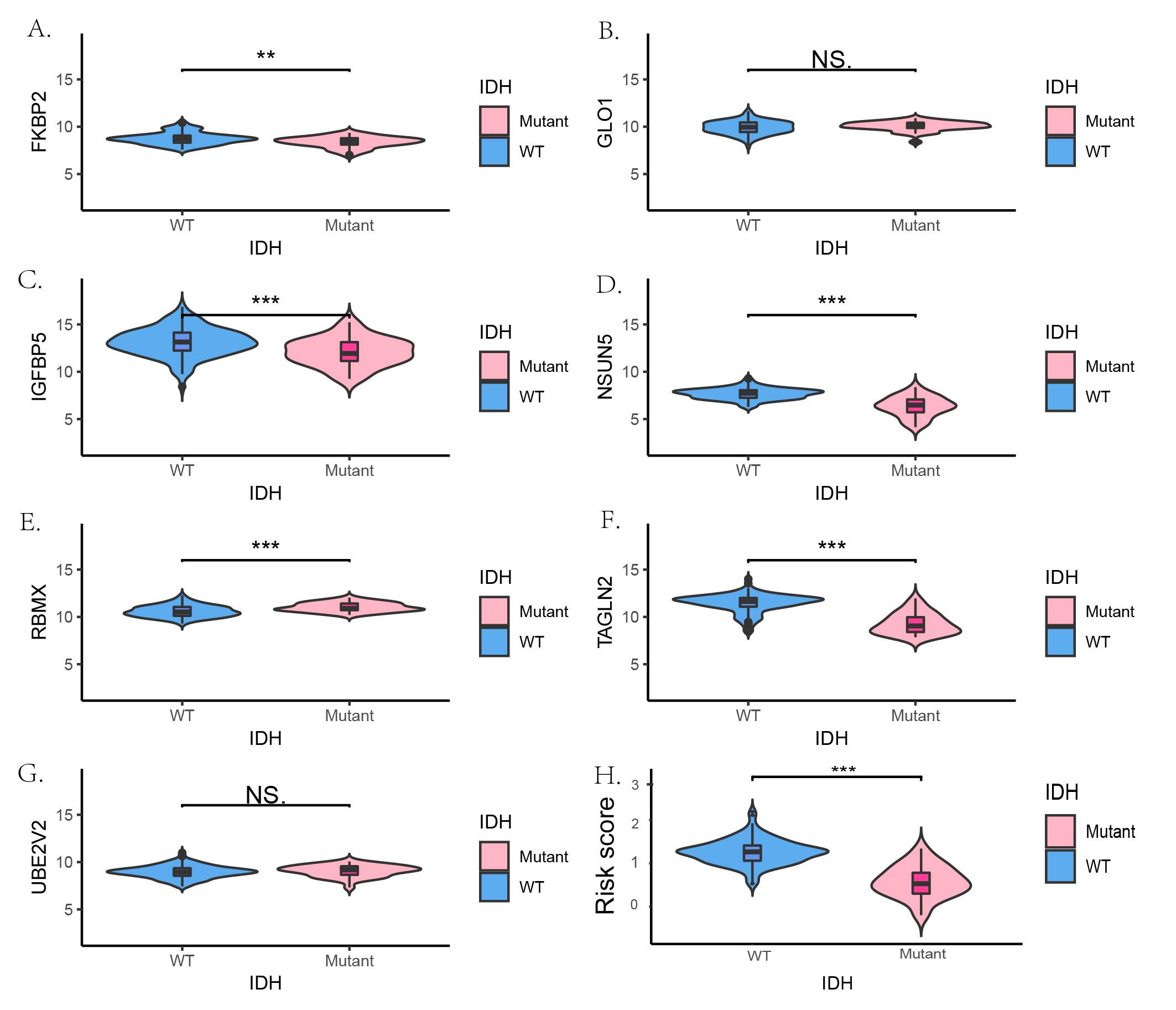

Supplement: Supplementary file 4 — Supplementary Figure 3. [file 41598_2021_95980_MOESM4_ESM.jpg]

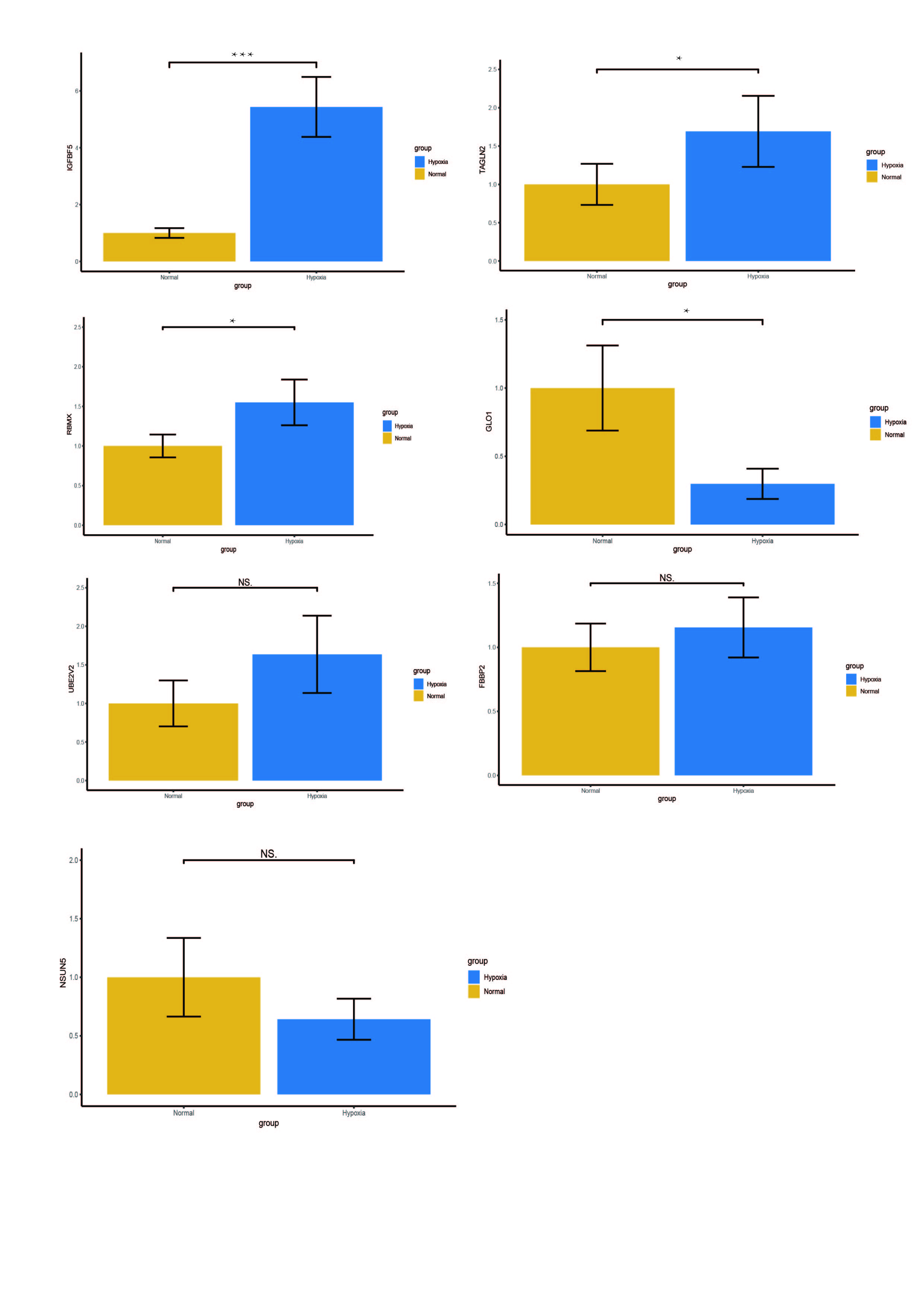

Supplement: Supplementary file 5 — Supplementary Figure 4. [file 41598_2021_95980_MOESM5_ESM.jpg]

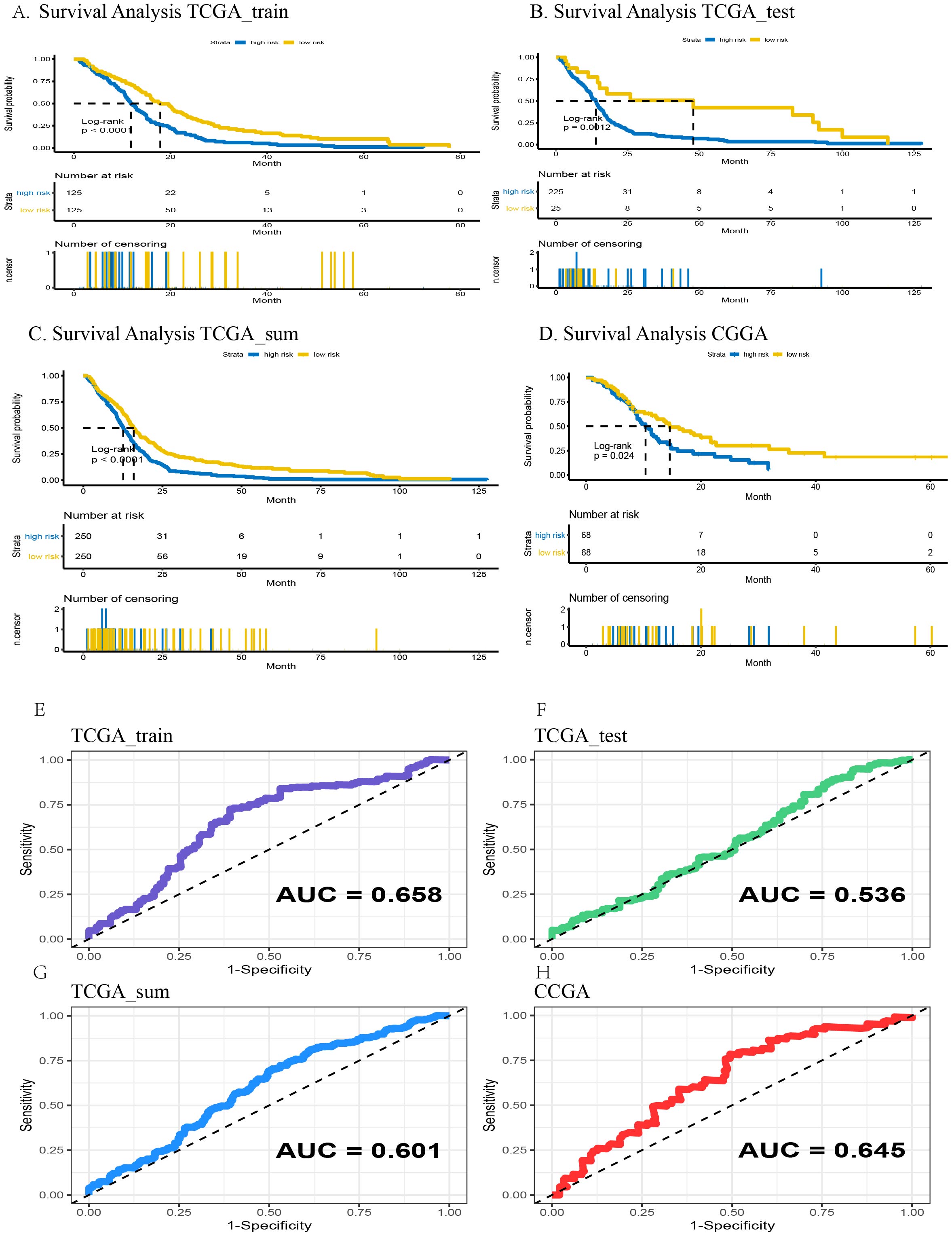

Supplement: Supplementary file 6 — Supplementary Figure 5. [file 41598_2021_95980_MOESM6_ESM.jpg]
