# Supplementary material for: Proteomics-based prognostic signature and nomogram construction of hypoxia microenvironment on deteriorating glioblastoma (GBM) pathogenesis
Source: Sci Rep. 2021 Aug 26;11:17170. doi: 10.1038/s41598-021-95980-x (PMC8390460; doi:10.1038/s41598-021-95980-x)
Supplement: Supplementary file 7 — Supplementary Table 1. [file 41598_2021_95980_MOESM7_ESM.docx]

**Supple. Table 1. Primer sequences list used in RT-PCR**

| Gene | primer sequences | Length (bp) |
| --- | --- | --- |
| Human-β-actin-F  Human-β-actin -R  Human-UBE2N-F  Human-UBE2N-R Human-FKBP-F  Human-FKBP-R Human-GLO1-F  Human-GLO1-R  Human-IGFBP5-F  Human-IGFBP5-R  Human-NSUN5-F  Human-NSUN5-R  Human-RBMX-F  Human-RBMX-R  Human-TAGLN2-F  Human-TAGLN2-R | TGGGCATGGAGTCCTGTG  TCTTCATTGTGCTGGGTG  CCTTTGAGGGAGGGACTT TCATCTGGATTGGGAGCA  GCCCTTTGTCTTCTCCCTT GCTCAGTTCGTCGCTCTATTTT  TTCTTGGAATGACGCTAA TTGCCATTGTGGTAACTCT  TGACCCAGTCCAAGTTTGTCG  CACCAGCAGATGCCACGT  GCCAGGAGGAGAATGAAGAC  CTCGACCCGTTCAATTACAG  CTCTTCATTGGTGGGCTTAA  GGTTTGGTGGCTTGTTCC  GACCTCTGGGAAGGAAAGA  GGTTGGTGCCCATCTGTA | 180  220  203  201  241  194  233  202 |
